# NDR Kinases Are Essential for Somitogenesis and Cardiac Looping during Mouse Embryonic Development

**DOI:** 10.1371/journal.pone.0136566

**Published:** 2015-08-25

**Authors:** Debora Schmitz-Rohmer, Simone Probst, Zhong-Zhou Yang, Frédéric Laurent, Michael B. Stadler, Aimée Zuniga, Rolf Zeller, Debby Hynx, Brian A. Hemmings, Alexander Hergovich

**Affiliations:** 1 Friedrich Miescher Institute for Biomedical Research, Basel, Switzerland; 2 Department of Biomedicine, Developmental Genetics, University of Basel, Basel, Switzerland; 3 Model Animal Research Center of Nanjing University, Pukou District, NanJing, P.R. China; 4 Swiss Institute of Bioinformatics, Maulbeerstrasse 66, Basel, Switzerland; 5 UCL Cancer Institute, University College London, London, United Kingdom; Northwestern University, UNITED STATES

## Abstract

Studies of mammalian tissue culture cells indicate that the conserved and distinct NDR isoforms, NDR1 and NDR2, play essential cell biological roles. However, mice lacking either *Ndr1* or *Ndr2* alone develop normally. Here, we studied the physiological consequences of inactivating both NDR1 and NDR2 in mice, showing that the lack of both *Ndr1/Ndr2* (called *Ndr1/2*-double null mutants) causes embryonic lethality. In support of compensatory roles for NDR1 and NDR2, total protein and activating phosphorylation levels of the remaining NDR isoform were elevated in mice lacking either *Ndr1* or *Ndr2*. Mice retaining one single wild-type *Ndr* allele were viable and fertile. *Ndr1/2*-double null embryos displayed multiple phenotypes causing a developmental delay from embryonic day E8.5 onwards. While NDR kinases are not required for notochord formation, the somites of *Ndr1/2*-double null embryos were smaller, irregularly shaped and unevenly spaced along the anterior-posterior axis. Genes implicated in somitogenesis were down-regulated and the normally symmetric expression of *Lunatic fringe*, a component of the Notch pathway, showed a left-right bias in the last forming somite in 50% of all *Ndr1/2*-double null embryos. In addition, *Ndr1/2*-double null embryos developed a heart defect that manifests itself as pericardial edemas, obstructed heart tubes and arrest of cardiac looping. The resulting cardiac insufficiency is the likely cause of the lethality of *Ndr1/2*-double null embryos around E10. Taken together, we show that NDR kinases compensate for each other *in vivo* in mouse embryos, explaining why mice deficient for either *Ndr1* or *Ndr2* are viable. *Ndr1/2*-double null embryos show defects in somitogenesis and cardiac looping, which reveals their essential functions and shows that the NDR kinases are critically required during the early phase of organogenesis.

## Introduction

The NDR (nuclear Dbf2-related) family of serine/threonine protein kinases represents a subclass of the AGC (protein kinase A (PKA)/PKG/PKC-like) kinases [1]. The mammalian genomes encode two highly related NDR kinases with 86% amino acid identity, called NDR1 and NDR2 (also termed STK38 and STK38L, respectively) [2–4]. Members of the NDR family are well conserved with important and in general essential roles in uni- and multi-cellular eukaryotes including yeast, fungi, plants, flies and mammals [1].

Studies in budding and fission yeast demonstrated that yeast NDR kinases are essential for survival of these unicellular organisms (summarized in [1]). Genetic inactivation of the *Drosophila* NDR kinase results in lethality during embryonic and/or larval development [5]. This lethality is rescued by expression of the human NDR1 kinase [6], suggesting that NDR kinases have conserved essential roles in multicellular organisms. However, *Ndr1*-deficient mice develop normally, are fertile and have normal life spans [7]. Similarly, it has been reported that mice deficient for *Ndr2* are born at the expected Mendelian frequency [8]. These findings are surprising considering the vital functions of NDR kinases in other species [1] and tissue culture-based experiments, which suggest that mammalian NDR kinases are essential for cellular processes such as centrosome duplication [9, 10], ciliogenesis [11], apoptosis [7, 12, 13] and cell cycle progression [14–19]. However in contrast to *Ndr1*-deficient mice [7], the currently available *Ndr2*-deficient mice [8] were generated by a gene-trap insertion in intron 9 of the murine *Ndr2* gene, which results in expression of a NDR2_1-282_::β-geo fusion protein [8]. Thus, analysis of complete loss-of-function *Ndr2* mice is needed to firmly establish if inactivation of *Ndr2* does really not alter viability. Therefore, to study the physiological importance of NDR1 and NDR2 kinases *in vivo*, we analyzed mice deficient for either *Ndr1* or *Ndr2* and double mutant mice. Significantly, our genetic analysis reveals that a single wild-type allele of *Ndr1* or *Ndr2* is able to sustain normal embryonic development, while complete inactivation *Ndr1* and *Ndr2* causes embryonic lethality. *Ndr1/2*-double null embryos displayed striking somite patterning defects and an early arrest in cardiac looping. The severe cardiac insufficiency appears to be the likely cause of the embryonic lethality at mid-gestation.

## Results

### *Ndr2*-deficient mice are phenotypically normal

**Fig 1:**
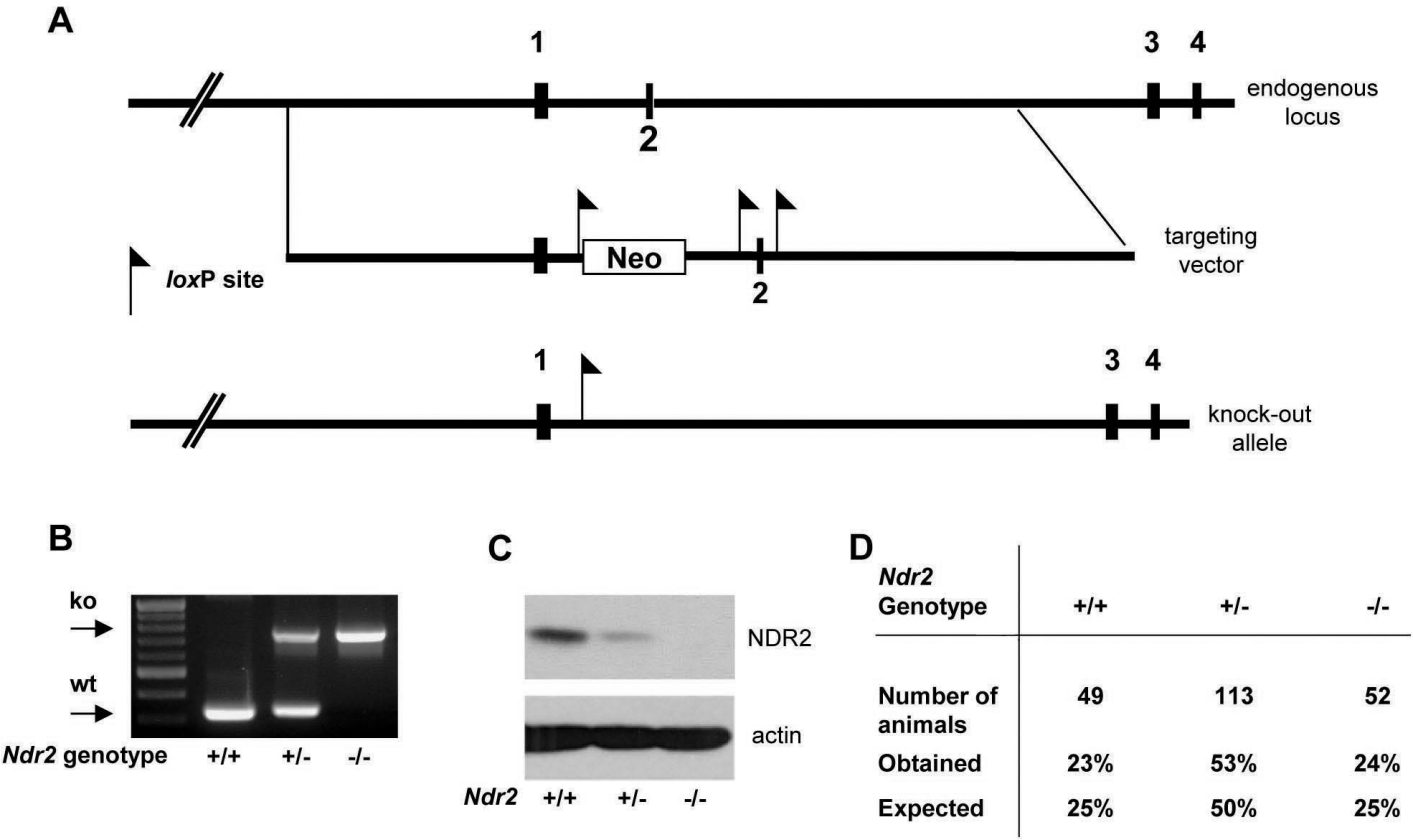
Generation and validation of *Ndr2* knock-out mice. (A) Genomic structure of the *Ndr2* locus in the mouse and targeting vector for *Ndr2* inactivation. Exons 1–4, and *Lox*P sites are indicated. Crosses with Cre-deleter mice removed exon 2, generating an *Ndr2* loss-of-function allele. (B) Genotyping of wild-type (+/+), heterozygous (+/-) and homozygous *Ndr2* knock-out (-/-) samples by PCR analysis. (C) Western blot analysis of NDR2 protein in colon lysates of wild-type (+/+), heterozygous (+/-) and homozygous *Ndr2*-deficient (-/-) littermates. (D) Genotype distribution of offspring from *Ndr2* heterozygous intercrosses. Genotypes were determined by PCR analysis at weaning.

*Ndr2*-deficient mice were generated by deleting coding exon 2 (Fig 1A), which encodes the *Ndr2* translation initiation codon [3]. Genotype analysis confirmed successful targeting of the *Ndr2* gene (Fig 1B; data not shown). Western blotting showed that the NDR2 protein was absent in *Ndr2*-deficient mice (Fig 1C). Similar to *Ndr1*-deficient mice [7], a gene-dosage effect was observed such that NDR2 protein levels already decreased in heterozygote mice (Fig 1C). As previously reported for *Ndr1*-deficient mice [7], and in full agreement with the analysis of *Ndr2* gene-trap allele [8], mice lacking the NDR2 protein were born at the expected Mendelian ratio, fertile and had a normal lifespan (Fig 1D; data not shown).

### Increased hydrophobic motif phosphorylation of the remaining NDR isoform in *Ndr*-single KO tissues

**Fig 2:**
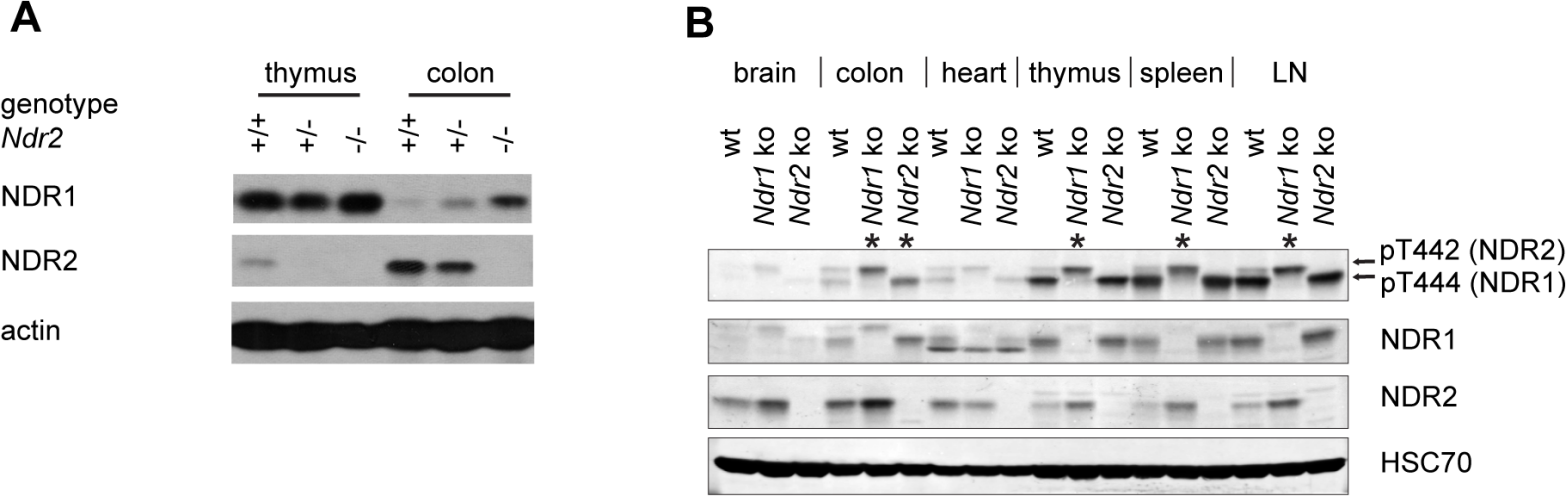
The remaining NDR isoform is upregulated in tissues of mice lacking one of the two *Ndr* genes. (A) Western blot analysis of NDR1 and NDR2 expression in thymus and colon of wild-type (+/+), *Ndr2* heterozygous (+/-) and *Ndr2*-deficient (-/-) mice. (B) Western Blot analysis of NDR1 and NDR2 proteins in brain, colon, heart, thymus, spleen and lymph nodes (LN) of wild-type (wt), *Ndr1*-deficient (ko 1) and *Ndr2* deficient (ko 2) mice. Hydrophobic motif phosphorylation, which serves as a direct indicator of NDR kinase activity, was examined using anti-phospho-T444/T442 antibodies (detecting NDR1 and NDR2 equally well [65]). The upper band corresponds to T442-P of NDR2, the lower band corresponds to T444-P of NDR1 [65]. HSC-70 served as loading control. Asterisks indicate compensatory HM phosphorylation events.

*Ndr1* and *Ndr2* display partially overlapping expression patterns and in all mouse tissues examined so far at least one of the two NDR isoforms is expressed [2–4, 7, 8]. While NDR1 protein levels are highest in organs of the immune system (thymus, spleen and lymph nodes), NDR2 protein levels peak in the colon and brain [2–4, 7, 8]. Intriguingly, NDR2 protein levels are post-transcriptionally up-regulated upon ablation of *Ndr1*, suggesting a compensatory link between the two NDR isoforms [7]. More precisely, up-regulation of NDR2 occurs particularly in tissues with high NDR1 expression in the wild-type situation [7]. To address whether the inactivation of *Ndr2* is also compensated by up-regulation of NDR1 protein, we analyzed thymus and colon tissue lysates of *Ndr2* wild-type, heterozygous and deficient adult littermate mice (Fig 2A). While NDR1 protein levels in the thymus of *Ndr2* heterozygous and deficient mice remained unchanged, NDR1 protein levels appeared to be increased in the colon of *Ndr2*-deficient mice (Fig 2A). This suggested that NDR1 compensates for loss of NDR2 in the colon, a tissue where NDR2 is normally highly expressed.

Using tissue culture systems, human NDR kinases were shown to play roles in centrosome duplication, apoptosis and cell cycle progression [20]. In all three processes, the hydrophobic motif (HM) phosphorylation of human NDR1 on Thr444 is essential, since rescue-experiments with the NDR1 T444A phospho-acceptor mutant did not compensate for loss of the wild-type protein [10, 12, 14, 19]. These studies indicated that Thr444/Thr442 phosphorylation is essential for, and reflects, NDR1/2 kinase activities [20]. Therefore, we determined whether the up-regulation of the remaining NDR isoform in single mutants tissues was paralleled by an increase in HM phosphorylation, which is a direct sensor of NDR kinase activity [20]. We detected apparently increased HM phosphorylation of NDR2 in the colon, thymus, spleen and lymph nodes of *Ndr1*-deficient mice, where NDR2 HM phosphorylation is normally low (Fig 2B). Conversely, NDR1 HM phosphorylation appeared to be elevated in the colon when *Ndr2* was inactivated (Fig 2B). In summary, our findings indicated that murine NDR1 and NDR2 may functionally compensate for each other *in vivo*, which would explain why mice lacking either *Ndr1* or *Ndr2* are viable and fertile.

### NDR kinases are essential for normal development after embryonic day E8

To address whether NDR kinases play an essential role during murine embryonic development *in vivo*, we generated mice lacking both *Ndr1 and Ndr2* (hereafter called *Ndr1/2*-double null). However, intercrossing *Ndr1* ^+/-^ and *Ndr2* ^+/-^ mice did not result in surviving *Ndr1/2*-double null mice (Table 1). *Ndr1* ^+/-^ *Ndr2* ^-/-^ and *Ndr1* ^-/-^ *Ndr2* ^+/-^ mice, which retained only one wild-type *Ndr* allele, were born fertile and did not display overt phenotypes (Table 1; data not shown), indicating that a single remaining wild-type allele is sufficient for normal development and reproduction, while complete loss of *Ndr1/2* results in embryonic lethality.

**Table 1:** *Ndr1/2*-double null mice are embryonic lethal, but a single *Ndr* allele is sufficient to sustain normal development.

*Ndr1* GT	*Ndr1*+/+	*Ndr1*+/+	*Ndr1*-/-	*Ndr1*-/-	*Ndr1*+/+	*Ndr1*+/-	*Ndr1*-/-	*Ndr1*+/-	*Ndr1*+/-
*Ndr2* GT	*Ndr2*+/+	*Ndr2*-/-	*Ndr2*-/-	*Ndr2*+/+	*Ndr2*+/-	*Ndr2*-/-	*Ndr2*+/-	*Ndr2*+/+	*Ndr2*+/-
**offspring numbers**	**37**	**23**	**0**	**28**	**44**	**54**	**42**	**53**	**134**
**theoretical (%)**	**6.25**	**6.25**	**6.25**	**6.25**	**12.5**	**12.5**	**12.5**	**12.5**	**25**
**actual (%)**	**8.92**	**5.54**	**0.00**	**6.75**	**10.60**	**13.01**	**10.12**	**12.77**	**32.29**

Genotype (GT) distribution of offspring from *Ndr-*single allele intercrosses (*Ndr1* ^+/-^;*Ndr2* ^-/-^ x *Ndr1* ^-/-^;*Ndr2* ^+/-^) at weaning. Actual offspring numbers are indicated, together with the expected and obtained Mendelian ratios. No *Ndr1/2*-double null mice were recovered. All other genotypes were obtained at approximately the expected Mendelian ratios. A total of 415 offspring were analyzed (n = 415).

**Fig 3:**
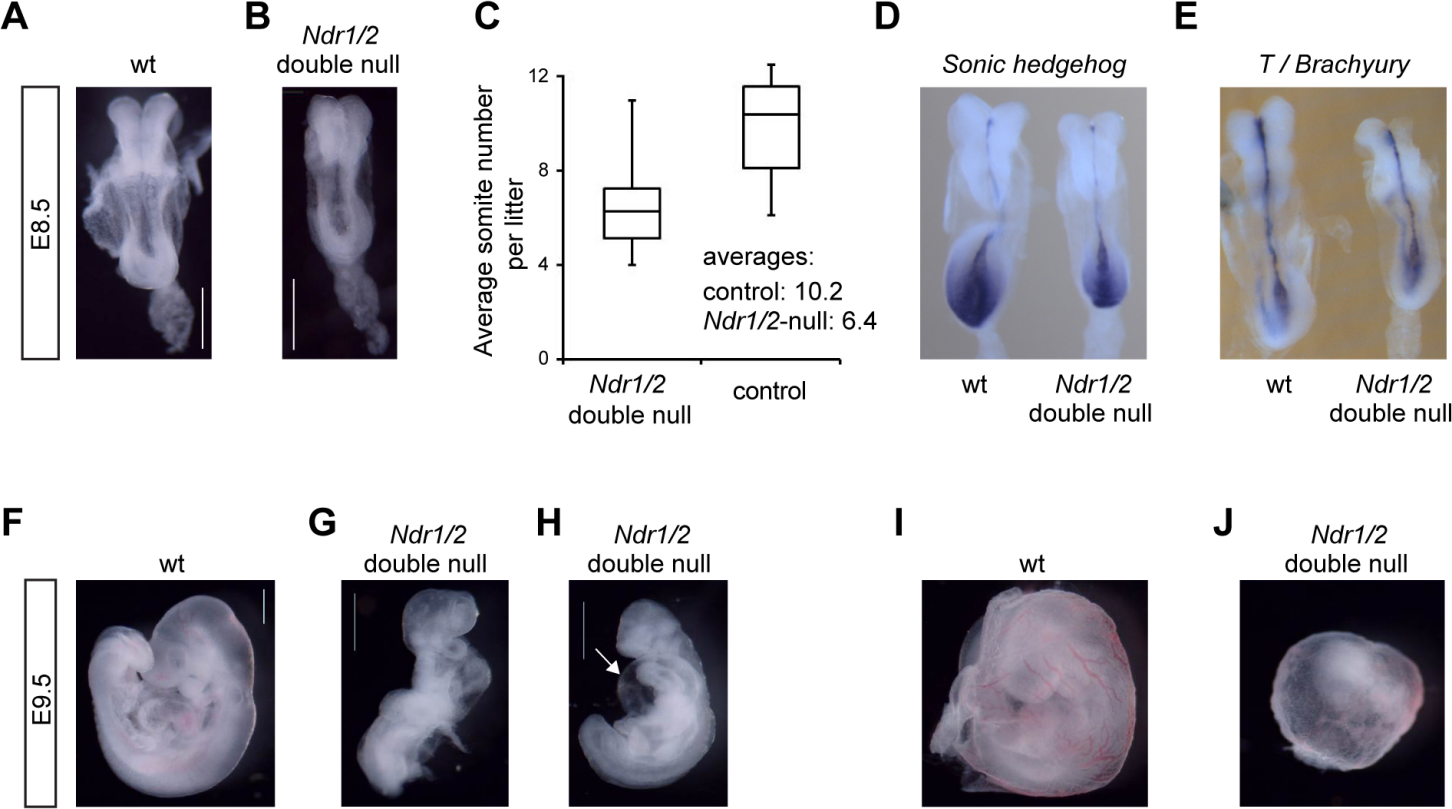
NDR kinases are essential for growth, cardiac development and blood vessel remodeling from about embryonic day 8 onward in mouse embryos. (A, B) Bright field images of wild-type (A) and *Ndr1/2*-double null (B) littermates at E8.5. Both embryos are of the 6-somite stage. Note that the *Ndr1/2*-double null somites are small and irregularly shaped. Scale bars correspond to 500μm. (C) Average somite numbers of wild-type and *Ndr1/2*-double null littermates at E8.5. Data correspond to the analysis of a total of 15 litters and are blotted as box and whisker chart illustrating the distribution of somite numbers per litter and genotype. Average somite numbers are indicated. (D, E) Distribution of *Shh* (D) and *T/brachyury* (E) transcripts in wild-type (left) and *Ndr1/2*-double null littermate embryos (right) at E8.5. Four animals per genotype were analyzed, and all embryos displayed the staining shown in Fig 3D and 3E. (F, G, H) Bright field images of wild-type (F) and *Ndr1/2*-double null (G, H) littermate embryos at E9.5. 56 *Ndr1/2*-double null and 163 control embryos at E9.5 were analyzed. White arrow in H points to the pericardial edema. Scale bars correspond to 500μm. (I, J) Bright field images of the yolk sacs of wild-type (I) and *Ndr1/2*-double null (J) littermate embryos at E9.5. All *Ndr1/2*-double null yolk sacs (n = 56) displayed defective vascular development as illustrated in Fig 3J.

To gain insight into the essential embryonic functions of NDR kinases, we analyzed *Ndr1/2*-double null embryos. At embryonic day 10.5 (E10.5), *Ndr1/2*-double null embryos suffered from severe growth retardation and were resorbed (Table 2; data not shown), indicating that NDR kinases are essential prior to this developmental period. At E10.5 all *Ndr1/2*-double null embryos isolated were dead (Table 2). Analysis of younger embryos revealed viable *Ndr1/2*-double null embryos at expected Mendelian ratio up to E9.5 (Table 2). However, already at E8.5 *Ndr1/2*-double null embryos appeared smaller and developmentally delayed as judged by their somite numbers (Fig 3A, 3B and 3C). Wild-type littermates had on average ten somites, while *Ndr1/2*-double null embryos had only six to seven somites at E8.5 (Fig 3C). Somites of *Ndr1/2*-double null embryos appeared also smaller and less defined (Fig 3B).

**Table 2:** *Ndr1/2*-double null embryos die around mid-gestation.

stage	*Ndr1*+/-	*Ndr1*+/-	*Ndr1*-/-	*Ndr1*-/-	unknown	total
	*Ndr2*+/-	*Ndr2*-/-	*Ndr2*+/-	*Ndr2*-/-		
**E9.5**	**53**	**54**	**56**	**56**	**7**	**226**
**E10.5**	**4**	**6**	**5**	**5** ^**a**^	**1**	**21**
**postnatal**	**69**	**65**	**59**	**0**	**0**	**193**

Genotype distribution in embryos derived from intercrosses of *Ndr1* ^+/-^;*Ndr2* ^-/-^ with *Ndr1* ^-/-^; *Ndr2* ^+/-^ mice at indicated time points. Note (a): all *Ndr1/2*-double null embryos recovered at E10.5 were dead and were in progress of resorption.

The notochord, a rod-like structure underlying the neural tube, participates in somite and neural tube patterning by secreting the Sonic Hedgehog (SHH) morphogen [21]. While SHH is essential to maintain the notochord [22], its formation depends on the T-box transcription factor T/Brachyury [23]. Therefore, we analyzed the expression of *Shh* (Fig 3D) and *T*/*Brachyury* in wild-type and *Ndr1/2*-double null embryos at E8.5 (Fig 3E). This analysis showed that the expression of both genes remained unaltered in *Ndr1/2*-double null embryos at E8.5, which pointed to normal notochord architecture and functions in *Ndr1/2*-double null embryos (Fig 3D and 3E). Four animals per genotype were analyzed, and all embryos displayed the staining shown in Fig 3D and 3E.

By E9.5, the size differences between wild-type and *Ndr1/2*-double null embryos were about two-fold (Fig 3F, 3G and 3H). Moreover, 50% of *Ndr1/2*-double null embryos had still not turned (Fig 3G), while wild-type embryos completed the turning process between E8.5 and E9.0 [24] (data not shown). Furthermore, approximately 50% of *Ndr1/2*-double null embryos had developed pericardial edema (Fig 3H) as indicated by excessive fluid accumulation in the heart region due to cardiac malfunction. Despite this impairment, cardiac contractions were observed in several *Ndr1/2*-double null embryos at E9.5 (data not shown). The yolks sacs of *Ndr1/2*-double null embryos also differed strikingly from their wild-type counterparts. In contrast to yolk sacs of wild-type embryos that had undergone extensive remodeling of the vascular plexus with readily detectable macroscopic blood vessels (Fig 3I), large remodeled vessels were absent from yolk sacs of *Ndr1/2*-double null embryos at E9.5 (Fig 3J). Specifically, at E9.5 all *Ndr1/2*-double null yolk sacs (n = 56) displayed defective vascular development as shown in Fig 3J (compare Fig 3I and 3J). Since some red blood cells were detected in yolk sacs of *Ndr1/2*-double null embryos, primitive hematopoiesis appeared to have occurred to a certain extent in the absence of NDR kinases. The allantois of *Ndr1/2*-double null embryos was well developed and attached to the chorion in all cases (data not shown).

### The CDK inhibitor p21/Cip1 is up-regulated in *Ndr1/2*-double null embryos

In the search for a possible molecular mechanism underlying the observed embryonic lethality, gene expression analyses comparing *Ndr1/2*-double null embryos with controls was done (S1 and S2 Tables). Although the phenotype was more severe at E9.5, the analyses focused on *Ndr1/2*-double null embryos at E8.5 in the hope to identify the primary defects caused by loss of the NDR kinases. Both *Ndr1* and *Ndr2* transcripts are broadly expressed at E8.5 as assessed by RNA *in situ* hybridization (S1 Fig). As embryonic development proceeds extremely fast, we focused our transcriptome analysis on embryos with seven to nine somites. The list of differentially expressed genes in *Ndr1/2*-double null embryos did not reveal any signature of apoptotic genes (S1 and S2 Tables). In line with these data, TUNEL analysis detected only few apoptotic cells in both wild-type and *Ndr1/2*-double null embryos at E8.5 (S2 Fig), indicating that the developmental delay was not due to increased apoptosis. Since in the intestinal epithelium of adult mice, NDR kinases were recently shown to regulate the growth promoting YAP proto-oncoprotein [25], we next investigated the expression of known YAP target genes in embryos [26] by comparing wild-type to *Ndr1/2*-double null embryos. However, this analysis revealed that the expression of YAP target genes is not altered in *Ndr1/2*-double null embryos at E8.5 (S3 Fig and S1 and S2 Tables).

**Fig 4:**
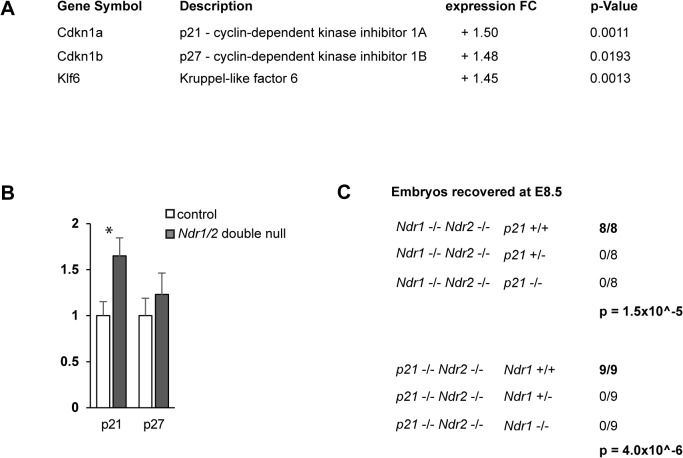
The CDK inhibitor p21/Cip1 is up-regulated in *Ndr1/2*-double null embryos and required for survival. (A) Changes in transcript levels between wild-type and *Ndr1/2*-double null embryos at E8.5 as revealed by microarray analysis (see S1 and S2 Tables). (B) Validation of the alterations in *p21* and *p27* expression in wild-type (control) and *Ndr1/2*-double null embryos by qRT-PCR (at E8.5). Data shown represent the average gene expression levels obtained by analyzing three independent embryos per genotype. Each embryo was analyzed in triplicate. Statistical analysis was performed using a two-tailed Student t-test assuming unequal variance. (C) Genotype distribution of offspring from *Ndr1* ^+/-^;*Ndr2* ^-/-^;*p21* ^+/-^ intercrosses. Embryos were isolated at E8.5 and genotyped. Top panel: *p21* genotype of the *Ndr1/2*-double null embryos; bottom panel: *Ndr1* genotype of the *p21*-null embryos. The p-values are indicative of the probabilities to obtain the observed genotype distribution by chance.

Intriguingly, the microarray analysis (S1 and S2 Tables) revealed that *Cdkn1a* and *Cdkn1b* transcripts, which encode the cyclin-dependent kinase (CDK) inhibitors p21/Cip1 and p27/Kip1, were up-regulated about 1.5 fold in *Ndr1/2*-double null embryos (Fig 4A). Quantitative real-time PCR analysis confirmed the up-regulation of p21 in *Ndr1/2*-double null embryos, while the increase in *p27* expression was not confirmed (Fig 4B). Moreover, *Krüppel-like 6* (*Klf6*) transcripts were elevated in *Ndr1/2*-double null embryos (Fig 4A). As the transcription factor Klf6 directly activates *p21* expression [27, 28], it is possible that their up-regulation is linked in *Ndr1/2*-double null embryos. Endogenous p21 has been shown to restrict cellular proliferation *in vivo* [29, 30], hence the up-regulation of p21 as cell cycle inhibitor indicates that the reduced size of *Ndr1/2*-double null embryos could be at least in part a consequence of impaired cell cycle progression.

In this regard, we analyzed the mitotic index of *Ndr1/2*-double null and wild-type embryos based on phospho-H3 staining, revealing that the mitotic index does not appear to be affected by complete *Ndr1/2* loss at E8.5 (S4 Fig). However, in a rapidly growing embryo, even minor changes in cell cycle duration may result in significant size differences. Thus, we developed a simple model to mathematically approximate the effect of increased cell cycle duration from E7.5 to E8.5 in mouse development (S5 Fig). This model revealed that a 20% increase in mean cell cycle duration during this period would suffice to generate the 1.5 fold size difference as observed between normal and *Ndr1/2*-double null littermates at E8.5 (Fig 3). Collectively, this suggests that the cell cycle progression in *Ndr1/2*-double null embryos deserves future investigations using highly sophisticated approaches.

### p21 becomes essential for embryonic development in *Ndr1/2*-double null embryos

To further investigate the potential contribution of the transcriptional up-regulation of p21 to the developmental arrest of *Ndr1/2*-double null embryos, we asked whether concomitant loss of p21 would rescue the growth defect of *Ndr1/2*-double null embryos. Previous analysis had established that p21 KO animals are viable and fertile and do not show any overt developmental phenotype [31]. However, to our surprise, we were unable to obtain mice carrying a single wild-type *Ndr1* single allele on a *p21*-deficient background (*Ndr1* ^+/-^;*Ndr2* ^-/-^;*p21* ^-/-^). In contrast, mice carrying one wild-type *Ndr1* single allele heterozygous for the *p21* loss-of-function (*Ndr1* ^+/-^;*Ndr2* ^-/-^;*p21* ^+/-^) were born at the expected Mendelian ratios (data not shown). These findings suggest that upon reduction of the *Ndr* gene dose to one functional copy, p21 becomes essential for normal embryonic development. We confirmed this finding by analyzing embryos from *Ndr1* ^+/-^;*Ndr2* ^-/-^;*p21* ^+/-^ intercrosses, which revealed that all of the eight *Ndr1/2*-double null (*Ndr1* ^-/-^;*Ndr2* ^-/-^) embryos isolated at E8.5 carried two wild-type *p21* alleles (Fig 4C, top). Conversely, all of the nine *p21*-deficient embryos recovered at E8.5 were wild-type for *Ndr1* (Fig 4C, bottom). Consequently, these findings point to an unexpected genetic dependency of *p21* and *Ndr1/2* kinases during embryonic development, hence additional studies are needed to gain insight into how the NDR- and p21-dependent signaling pathways interact during embryonic development.

### Analysis of abnormal somite development in *Ndr1/2*-double null embryos

**Fig 5:**
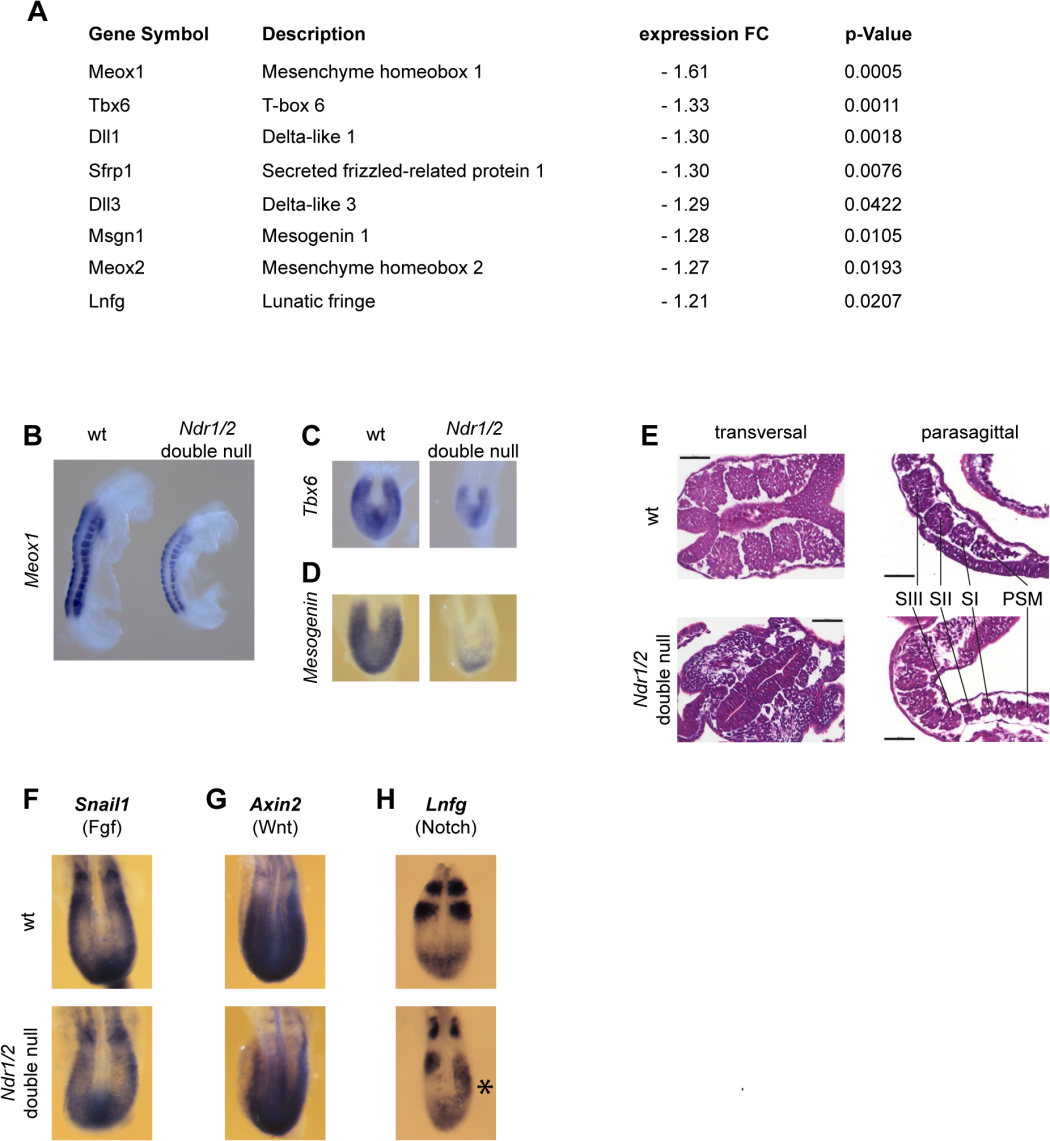
Somitogenesis is altered in *Ndr1/2*-double null embryos. (A) Changes in gene expression between wild-type and *Ndr1/2*-double null embryos at E8.5 determined by microarray analysis (see S1 and S2 Tables). (B, C, D) Distribution of *Meox1* (B), *Tbx6* (C) *and Mesogenin1* (D) transcripts in wild-type (wt) and *Ndr1/2*-double null embryos at E8.5. Four animals per genotype were analyzed for *Meox1* staining, and two embryos per genotype were analyzed for *Tbx6* and *Mesogenin* staining. All embryos displayed the staining patterns as shown in Fig 5B, 5C and 5D. (E) Hematoxylin/Eosin staining of transversal (left) and parasagittal (right) sections of wild-type (top panels) and *Ndr1/2*-double null (bottom panels) embryos at the 6-somite stage. Six embryos of each genotype were analyzed. PSM: presomitic mesoderm; SI, SII, SIII: last, second to last and third to last formed somite. Scale bars = 50μm. (F, G, H) Distribution of *Snail1* (F), *Axin2* (G) *and Lnfg* (H) transcripts in wild-type (wt) and *Ndr1/2*-double null embryos. Asterisk (*) indicates aberrant asymmetrical expression (right) of *Lnfg* in the last formed somite pair. Seven embryos were analyzed per genotype for *Snail1* staining, and five embryos were analyzed per genotype for *Axin2* staining. The expression patterns of *Snail1* and *Axin2* appeared indistinguishable between all mutant and control embryos. Nine mutant and four control embryos were analyzed for *Lnfg* staining. All four control embryos showed the expected symmetric expression pattern as illustrated in Fig 5H. In contrast, four of nine mutant embryos displayed strongly asymmetric *Lnfg* expression and one mutant embryo showed mild asymmetric expression.

The microarray data also revealed a set of genes predominantly expressed in somites or being implicated in somitogenesis that were down-regulated in *Ndr1/2*-double null embryos, which includes the transcription factors *Meox1*, *Meox2*, *Tbx6* and *Mesogenin1* (Fig 5A). *Mesogenin1* is essential for presomitic mesoderm maturation [32], *Meox1* and *Meox2* are essential for normal somitogenesis [33], and reduction of *Tbx6* disrupts somite patterning [34, 35]. Therefore the expression of these genes was analyzed by whole mount RNA *in situ* hybridization in E8.5 wild-type and *Ndr1/2*-double null embryos (Fig 5B to 5D). In wild-type embryos (Fig 5B, left embryo), *Meox1* expression demarcated the somite boundaries and revealed the even distances between somite pairs. In *Ndr1/2*-double null embryos (Fig 5B, right embryo), *Meox1* expression was decreased, somite borders appeared fuzzy, distances between somites varied and the positions of the last formed somites was not well defined. Analysis of the *Tbx6* and *Mesogenin1* expression patterns confirmed their down-regulated expression in *Ndr1/2*-double null embryos (Fig 5C and 5D). Analysis of somite morphology revealed that the size of *Ndr1/2*-double null somites was significantly reduced (Fig 5E, six somites stage at E8.5). In summary, *Ndr1/2* deficiency results in transcriptional down-regulation of genes involved in somite formation by E8.5. Somites of *Ndr1/2*-double null embryos were smaller, irregularly shaped and unevenly spaced along the anterior-posterior (AP) embryonic axis, which indicates that NDR kinases function in somite formation and spacing.

### Asymmetric expression of the Notch pathway component *Lunatic Fringe* in the last formed somite in *Ndr1/2*-double null embryos

Somitogenesis is a highly symmetrical process such that the left and the right somite of each pair form together. The so-called segmentation clock coordinates somite formation by ensuring the formation of somite pairs in a periodic fashion (reviewed in [36]). The segmentation clock is operated by cyclic expression of distinct members of the FGF, Wnt and Notch pathways [36]. Interestingly, we noted that the expression of the WNT antagonist *Sfrp1* (secreted frizzled-related protein 1) was decreased in *Ndr1/2*-double null embryos (Fig 5A). In addition, the expression of three components of the Notch signaling pathway was also reduced in *Ndr1/2*-double null embryos, namely *Lunatic fringe* (*Lnfg*) and the Notch ligands *Delta-like Dll1* and *Dll3* (Fig 5A). *Sfrp1* was previously shown to regulate A-P axis elongation and somite segmentation in conjunction with *Sfrp2* in mouse embryos [37]. LNFG negatively regulates Notch signaling and *Lnfg*-null mice display defects in somitogenesis [38], while Dll1 and Dll3 are essential for somite formation [39, 40] and establishment of the inter-somitic boundaries [41]. Thus, the deregulation of these genes in *Ndr1/2*-double null embryos could be cause or consequence of the observed somitogenesis defects. Therefore, the expression of the *Snail1* [42], *Axin1* [43] and *Lnfg* genes was analyzed during somitogenesis (Fig 5F, 5G and 5H). The expression patterns of *Snail1* (Fig 5F) and *Axin2* (Fig 5G) were indistinguishable between all mutant and control embryos. In contrast, the *Lnfg* expression, which is a negative regulator of the Notch pathway, was altered in *Ndr1/2*-double null embryos at E8.5 (Fig 5H). In contrast to its normal symmetrical expression in wild-type embryos (Fig 5H, top panel), *Lnfg* expression was asymmetric in the last formed somite pair in 50% of *Ndr1/2*-double null embryos (Fig 5H, bottom panel). The asymmetric *Lnfg* expression pattern suggests that formation of the last somite pair occurred in a temporally discoordinate fashion. This indicates that NDR kinases are required for symmetric *Lnfg* expression and bilaterally coordinated somite formation (see discussion).

### NDR kinases are essential for cardiac looping during mouse embryonic development

Aberrant somitogenesis is unlikely to account for embryonic lethality since heart development is not disrupted in mouse embryos lacking *Meox1/2*, which severely disrupts somites and results in the absence of the axial skeleton [33]. The heart is essential for survival for the embryo from early organogenesis onward [44]. The primitive heart tube forms around E8.0 [45, 46], begins to rhythmically contract at around the 3-somite stage and the onset of blood flow commences around 4 to 6 somites [47]. The development of the linear heart tube into the four-chambered adult heart occurs by cardiac looping and is the first morphologically distinct asymmetric structure during organogenesis. Cardiac looping is initiated between E8.0 and E8.5 [48] and is categorized into the heart looping stages (LS) zero to III [49]. At LS-0, the A/P axis of the heart is parallel with that of the neural tube and pharynx. At LS-I, the heart tube starts to display asymmetric development by adopting a tilted A/P axis in the left/right plane. At LS-II, a separate atrial chamber and atrioventricular sulcus are first apparent in the heart tube, while looping of the ventricular region is yet absent or minimal. At LS-III, overt ventricular looping begins together with asymmetrical atrial development. At this stage, the left ventricle expands, thereby occupying space in front of the atrial chamber and left sinus venosus.

**Fig 6:**
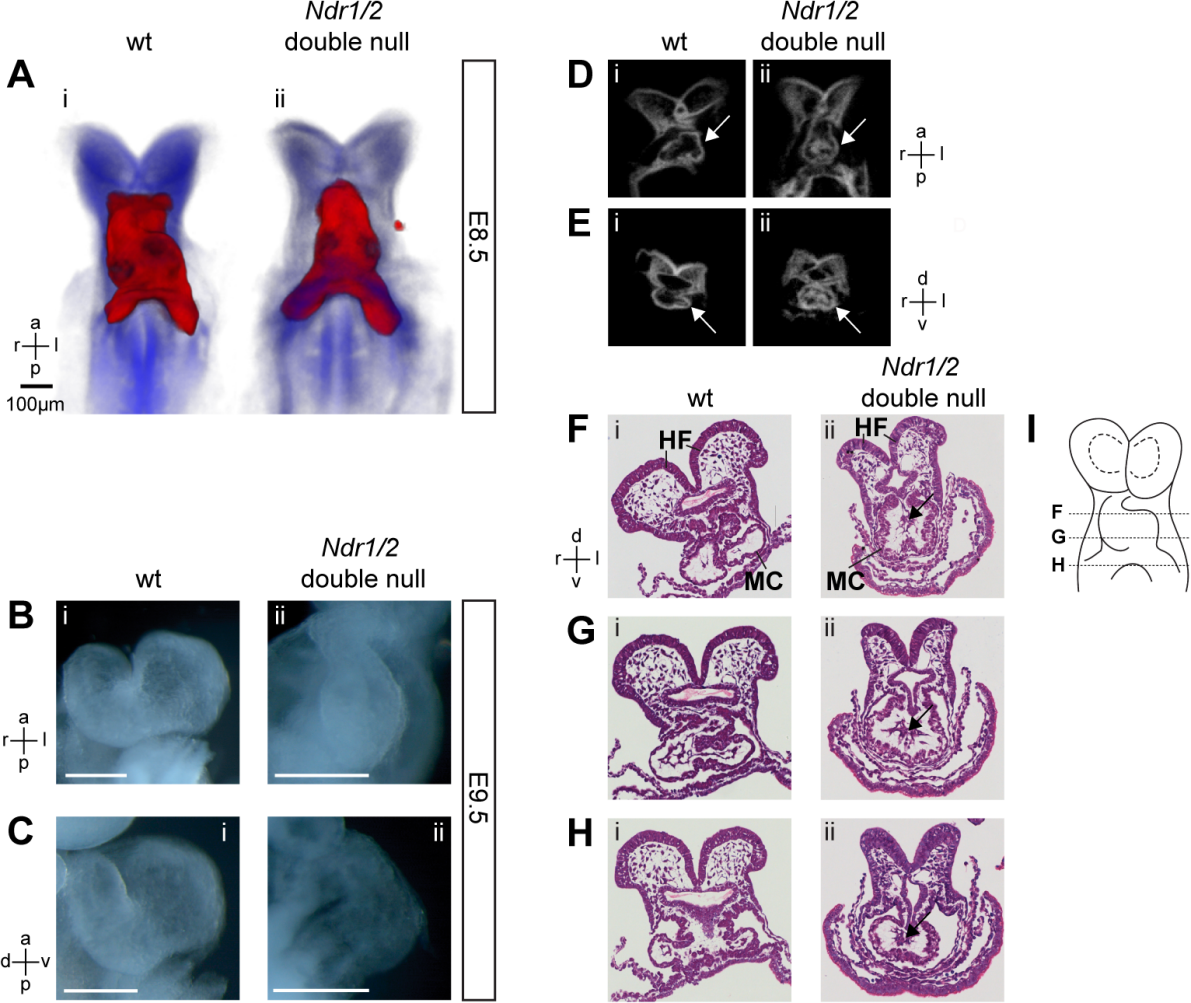
Murine NDR kinases are essential for cardiac looping. (A) OPT 3D reconstruction of wild-type (Ai) and *Ndr1/2*-double null (Aii) embryos at E8.5. Blue: anatomy. Red: *Nkx2*.*5* whole mount *in situ* hybridization. Embryo axis orientation: a: anterior, p: posterior, l: left, r: right. Two *Ndr1/2*-double null and two control embryos were examined. Using bright field microscopy ten *Ndr1/2*-double null and ten control embryos were analysed at E8.5 to confirm the observed phenotype (data not shown). (B, C) Bright field images of wild-type (Bi, Ci) and *Ndr1/2*-double null (Bii, Cii) developing hearts at E9.5. B: frontal view; C: lateral view. Scale bars = 100μm. Embryo axis orientation: a: anterior, p: posterior, l: left, r: right, d: dorsal, v: ventral. Five *Ndr1/2*-double null and five control embryos were analyzed. (D, E) OPT virtual section of wild-type (Di, Ei) and *Ndr1/2*-double null (Dii, Eii) embryos shown in 6A. Panel D: coronal plane; panel E: transversal plane. Labels: a: anterior, p: posterior, d: dorsal, v: ventral. Arrows point to the heart. Two *Ndr1/2*-double null and two control embryos were analyzed for OPT as shown in 6D and 6E. (F, G, H) Hematoxylin and Eosin stained transversal sections of a wild-type (Fi, Gi, Hi) and *Ndr1/2*-double null (Fi, Gi, Hi) hearts at the 6-somite stage. The myocardium (MC) and headfolds (HF) are indicated in (Fi) and (Fii). Arrows in (Fii), (Gii) and (Hii) point to remaining cells in the cardiac jelly and lumen. Note the similar section plan between the embryos shown in (E) and (G). Three *Ndr1/2*-double null and three control embryos were analyzed. (J) Scheme showing the approximate level of the sections within the embryo. The distance between sections is about 30μm.

Comparative analysis of developing hearts at E8.5, showed that while wild-type hearts had progressed to LS-III with prominent rightward looping (Fig 6Ai), the looping of *Ndr1/2*-double null hearts remained in stage LS-II (Fig 6Aii). In addition, *Ndr1/2*-double null hearts displayed a bulbous morphology and appeared less transparent than wild-type controls (data not shown), which pointed to either a thickened myocardium or a reduced heart lumen. To determine whether this striking heart phenotype was due to a developmental delay or arrest, E9.5 embryos were analyzed (Fig 6B and 6C). While the looping process had progressed in wild-type hearts (Fig 6Bi and 6Ci), the heart looping of *Ndr1/2*-double null embryos remained arrested at LS-II at E9.5 (Fig 6Bii and 6Cii). In agreement with this observation, no rightward looping of *Ndr1/2*-double null embryonic hearts was observed. In addition, *Ndr1/*2-double null embryos appeared smaller than their wild-type litter mates at E9.5, but organogenesis was not arrested at this stage (Fig 3). OPT (Optical Projection Tomography) analysis established that the lumen of the developmentally arrested *Ndr1/2*-double null embryonic hearts had not formed properly (Fig 6D and 6E). Indeed, analysis of histological sections revealed that the myocardium of *Ndr1/2*-double null embryonic hearts was thickened (Fig 6Fii, 6Gii and 6Hii) and the cardiac jelly and/or lumen contained additional cells (indicated by arrows in Fig 6Fii, 6Gii and 6Hii). Taken together, the pericardial edema (Fig 3H), arrest of cardiac looping (Fig 6A, 6B and 6C) and defects in the heart lumen (Fig 6D to 6H) indicated that these cardiac defects are the likely cause of the embryonic lethality of *Ndr1/2*-double null embryos during early mouse organogenesis.

## Discussion

Here, we investigated the essential roles of the murine NDR1 and NDR2 kinases. As previously reported for *Ndr1*-deficient mice [7], mice lacking *Ndr2* develop normally and are fertile. This is likely due to functional compensation as the levels and HM phosphorylation of the other NDR isoform were up-regulated in mice lacking only one of the two *Ndr* genes (this study and ref. [7]). We now establish that complete genetic inactivation of both *Ndr* genes in mice causes embryonic lethality around E10. In particular, our analysis establishes that NDR kinases are essential for mouse embryonic heart and somite development. The small and irregular somite morphology in *Ndr1/2*-double null embryos is accompanied by downregulation of genes functioning in somitogenesis such as *Meox1/2* [33]. However, the observed somite defects observed in *Ndr1/2*-double null embryos are less severe than the ones observed following genetic inactivation of *Lnfg* and the Notch Delta-like ligands *Dll1*, *Dll3* in mice [38–40]. Interestingly, the normally symmetric expression of *Lnfg* transcripts becomes asymmetric in the last formed somite pair in *Ndr1/2*-double null embryos. The unilateral posterior expanded expression of *Lnfg* is indicative of temporal heterochrony in the formation of left and right somites. This indicates that NDR kinases are part of the mechanism by which somites normally escape the already existing left-right asymmetry along the primary embryonic axis [36, 50]. Possibly NDR kinases play a role in the conserved ‘clock-and-wavefront’ mechanism controlling somitogenesis [51] and/or also contribute to the retinoic acid-mediated coordination of somitogenesis and left-right patterning [52–55]. Therefore, future research into the underlying molecular and cellular mechanisms is warranted.

Additional evidence in favor of a possibly more general role of NDR kinases downstream of establishing L/R asymmetry [36] is their essential role during cardiac looping [46, 56], which arrests at an early stage in *Ndr1/2*-double null embryos (this study). In addition, the thickened myocard and remaining cells in the cardiac jelly and lumen will likely interfere with normal blood flow and hamper normal embryo growth and organogenesis of *Ndr1/2*-double null embryos, resulting in their death during mid-gestation. Since remodeling of the yolk sac vasculature depends on hemodynamic forces exerted by blood flow [47] the observed defects in the yolk sac vasculature are likely secondary to the heart defects in *Ndr1/2*-double null embryos.

Much progress has been made in deciphering the transcriptional networks that govern the patterning of the vertebrate heart [56]. However, we did not identify changes in the expression of major regulators of cardiac development in *Ndr1/2*-double null embryos at E8.5, therefore the underlying molecular alteration resulting in arrest of cardiac looping remain unknown. In general, the mechanism underlying cardiac looping are not well defined [49, 57], but studies indicate that changes in myocardial cell shape, actin re-arrangements and extra-cellular matrix remodeling are key events during cardiac looping [58–61]. In this context, it is noteworthy that over-expressed human NDR2 associates with actin [4] and that one of the yeast NDR kinases interacts with the actin cytoskeleton [1]. Thus, the murine NDR kinases might function during cardiac looping by impacting on the underlying cytoskeleton re-arrangements. Consequently, more in depth molecular analysis will be needed to gain a better understanding of the possibly common and/or distinct roles of NDR kinases during mouse embryonic somitogenesis and cardiac looping.

It has been previously established that MST1/2 kinases function as direct upstream kinases of NDR1 and NDR2 in mammalian cells in culture [10, 12]. The genetic analysis of the *Mst1/2* [62–64] and *Ndr1/2* kinases in mice (this study) reveals striking similarities with respect to their genetic requirement and phenotypes: (a) a single wild-type allele of either *Ndr1/2* or *Mst1/2* is sufficient to sustain normal embryonic development, while complete inactivation of either the *Ndr1/2* or *Mst1/2* genes causes mid-gestational embryonic lethality; (b) *Ndr1/2*-double null and *Mst1/2*-null embryos are growth-retarded and developmentally delayed by E8.5; and (c) both types of null embryos display pericardial edema and a defective yolk sac vasculature (this study and refs. [62–64]). These similarities in embryonic mutant phenotypes is in agreement with the notion that NDR kinases might act as down-stream effectors of MST1/2 signaling during mouse embryonic development.

Taken together, our genetic analysis points to essential functions of mouse NDR kinases during early embryonic growth, temporally and spatially symmetric formation of somite pairs, early heart looping and chamber development.

## Materials and Methods

### Ethics statement (for animal experiments)

All studies with mice were approved by the Swiss Cantonal Veterinary Office of both Basel and Argovia. All mouse experimental analysis was performed in strict compliance with the animal welfare and ethic regulations and 3R principles as defined by the Swiss Federal laws governing animal research (Animal Welfare Act; Animal Welfare Ordinance; Animal Experimentation Ordinance).

### Study design

To investigate the role of murine NDR kinases in embryonic development, we compared *Ndr1/2*-double null mouse embryos and control littermates ranging in developmental age from E8.5 to E10.5 using different approaches, namely gross morphological analyses via bright field microscopy, RNA *in situ* hybridization, histological analyses (H&E and TUNEL), Optical Projection Tomography (OPT), microarray analyses and quantitative real-time PCR. All methods are described in detail in the respective sections below.

### Animal experiments

Results described here were from *ex-vivo* analysis of embryos post-mortem. Timed mating of naïve mice were performed to calculate correct Embryo age. When possible after timed mating, females were group housed. Breeding strategy for analysis of DKO embryonic lethality was optimized for DKO generation with single KO controls. None of the animals received study specific treatment. To obtain embryos, pregnant females were sacrificed by cervical dislocation prior to removal of embryos.

### Targeting of the murine *Ndr2* locus and mouse breeding

We introduced *lox*P sites up- and downstream of exon 2 of *Ndr2* via homologous recombination in Ola129 ES cells. Two independent ES cell clones (validated by Southern blotting, PCR and DNA sequencing) were expanded and aggregated with E2.5 morulas followed by re-implantation into pseudo-pregnant foster mothers. Chimeric offspring were crossed with the Meox2-Cre delete strain (B6.129S4-*Meox2* ^*tm1(cre)Sor*^/J) to delete *Ndr2* constitutively (B6CF2.129P2-*NDR2* ^*tm1/BAH-FMI*^). *Ndr1* loss-of-function (B6.129P2-*NDR1* ^*tm/BAH-FMI*^) mice have been described previously [7]. Mice were housed in Optimal Hygiene (Conventional) Conditions in Ventirack cages on Lignocel BK8-15 bedding with Rodent chow and water *ad libitum* and cage changes performed in Change Stations. Room conditions are monitored and maintained at 22+/-2°C temperature and 45–65% humidity with a 12 hour light/dark cycle. Kleenex tissue or nestlets are provided for enrichment. Mice are routinely group housed. Housing capacity is dependent upon cage type and mouse size. Mice are routinely monitored for ill-health/injury/adverse effects. Phenotypes of viable NDR GM mice have been assessed and do not require special conditions or interventions. Breeding strategy for analysis of *Ndr1/2*-double null (B6.129P2-*NDR1* ^*tm/BAH-FMI*^ *NDR2* ^*tm1/BAH-FMI*^) embryonic lethality was optimized for DKO generation with single KO controls.

### Sample sizes for animal experiments

Numbers of embryos analyzed and replicates performed to generate each data point are as follows: for gross morphological analysis (Fig 3), 15 litters with *Ndr1/2*-double null and control embryos at E8.5 as well as 56 *Ndr1/2*-double null and 163 control embryos at E9.5 were analyzed; for WISH (Figs 3 and 5); four animals per genotype were analyzed for *Shh* and *T*/*Brachyury* staining (Fig 3D and 3E), four animals per genotype were analyzed for *Meox1* staining (Fig 5B), two embryos per genotype were analyzed for *Tbx6* and *Mesogenin* staining (Fig 5C and 5D), seven embryos were analyzed per genotype for *Snail1* staining (Fig 5F), five embryos were analyzed per genotype for *Axin2* staining (Fig 5G), and nine *Ndr1/2*-double null and four control embryos were analyzed for *Lnfg* staining (Fig 5H); for H&E stainings of somite sections (Fig 5E) six *Ndr1/2*-double null and six control embryos were analyzed; for gross morphological analysis of the heart (Fig 6C and 6D) five *Ndr1/2*-double null and five control embryos at E9.5 were analyzed; for OPT analyses (Fig 6A, 6D and 6E) two *Ndr1/2*-double null and two control embryos were examined; and for H&E stainings of heart sections (Fig 6F, 6G, and 6H) three *Ndr1/2*-double null and three control embryos were analyzed. One representative series of analysis is shown for each *Ndr1/2*-double null and control embryos. Unless otherwise stated in the main text and/or figure legends, all embryos analyzed displayed the phenotypes/staining shown in the corresponding figure panels.

### Genotyping

The wild-type and loss-of-function *Ndr2* alleles were distinguished using a common forward primer: ^5’^gctgggataggtggataaatgg^3’^ and the following reverse primers: ^5’^gcttaagtcttaagctcaacctc^3’^ for wild-type yielding a PCR product of 424 bp and ^5’^gcctgcattgcagtccttagc^3’^ for the loss-of-function allele yielding a PCR product of 843 bp. Genotyping of the wild-type and loss-of-function *Ndr1* alleles was done as described [7].

### Antibodies and immunoblotting

The following rabbit polyclonal antibodies were used for Western blotting: total NDR1 and NDR2 [7]; phospho-T444/T442 [65]. The anti-actin antibody (sc-1616) was obtained from Santa Cruz, the HSC70 antibody (Clone 1B5) from Stressgen. Western blotting analysis was done as described previously [7].

### Microarray analysis

Microarray analysis used total mRNA prepared from male mouse embryos at the 7–9 somite stage. The pooled mRNAs from three *Ndr1/2*-double null and control littermates were compared. RNA was processed using the WT cDNA Synthesis & Amplification kit and cDNAs labeled using the WT Terminal Labeling kit from Affymetrix (Affymetrix, Santa Clara, CA) according to manufacturer's instructions. GeneChip Mouse Gene 1.0 ST arrays were hybridized according to the "GeneChip Whole Transcript (WT) Sense Target Labeling Assay Manual" (Affymetrix, Santa Clara, CA). The Affymetrix Fluidics protocol FS450_0007 was used for washing. Scanning was done using the Affymetrix GCC Scan Control v. 3.0.1 on a GeneChip Scanner 3000 with autoloader (Affymetrix). Probe sets were summarized and probe set-level values normalized with the “justRMA” function from R (version 2.10.0); the Bioconductor (version 2.5) package using the CDF environment MoGene-1_0-st-v1.r3.cdf (as provided by Bioconductor) and annotated from Netaffx (www.netaffx.com). Differentially expressed genes were identified using the empirical Bayes method (F test) as part of the LIMMA package and adjusted with the false discovery rate method as described [66]. Hierarchical clustering and visualization was done in R. Probe sets with a log2 average contrast signal of at least 5, a P value of <0.05, and an absolute log2 fold-change of >0.263 (1.2-fold in linear space) were selected. This lead to the identification of 701 transcriptionally up-regulated and 183 down-regulated genes in *Ndr1/2*-double null embryos (S1 and S2 Tables).

### Quantitative Real-time PCR (qRT-PCR)

qRT-PCR was performed using RNA extracted from embryos (see above). cDNA was generated from 1μg of total RNA using the M-MuLV reverse transcriptase (NEB) and random hexamer primers. Quantitative RT-PCR to detect *p21*, *p27*, *Ctgf*, *Acta2* and *Tagln* expression levels was done using SYBR green in an ABI Prism 7000 detection system (Applied Biosystems). The primer sequences for amplifying *Ctgf*, *Acta2* and *Tagln* were taken from ref. [43]. Primer sequences for *p21* and *p27* were obtained from the Harvard Primer Bank. All data shown are the average of analyzing three independent embryos per genotype. To determine the statistical significance of the observed differences in gene expression, a two-tailed T-test was performed assuming unequal variance.

### Whole-mount *in situ* hybridization and TUNEL analysis

Whole-mount *in situ* hybridization was performed according to ref. [67]. The following DIG-labeled riboprobes were used to detect transcripts: *Axin2* [43]; *Lunatic Fringe* [68]; *Meox1* [69]; *Mesogenin1* [32]; *Snai1* [70]*; Sonic hedgehog* [71]; *T/brachyury* [72]; *Tbx6* [73]. *Ndr1* and *Ndr2* riboprobe were generated by PCR using mouse cDNA and the primers: ^5’^cgatatctattgaaatcaagag^3’^ and ^5’^ttccccttcattctgatcaacttg^3’^ (for *Ndr1*; amplifying the nucleotides corresponding to 1416 to 1930 of NM_134115); and ^5’^ggaaagaccagcagctattcc^3’^ and ^5’^tgcagttctggctggattagtg^3’^ (for *Ndr2*; amplifying the nucleotides corresponding to 1305 to 1850 of NM_172734). TUNEL analysis was performed on paraffin sections with the ApoAlert DNA Fragmentation Assay Kit (Clontech) as defined by the manufacturer using the Venata Biobench machine.

### Optical Projection Tomography

Optical Projection Tomography (OPT, [74]) was used to generate virtual optical sections of wild-type and *Ndr1/2*-double null embryos. Samples were fixed overnight in 4% PFA, 0.2% glutaraldehyde in PBS and washed extensively. Then they were embedded in 1% low melting point agarose (Sigma), dehydrated in methanol and cleared in benzyl alcohol-benzyl benzoate. An OPT 3001M scanner (Bioptonics, MRC Technology) was used to acquire high-resolution images (1024x1024 pixels) of the sample anatomy using the GFP1 filter (425/40nm, 475nm LP). No filter (bright field) was used to acquire whole mount *in situ* hybridization signals. SkyScan software was used to scan the sample and NRecon software to reconstruct the 3D topology. Virtual sections of the 3D reconstructed embryos were obtained using the DataViewer software.

## Supporting Information

S1 ARRIVE Checklist Completed “ARRIVE Guidelines Checklist” for reporting data regarding animal research in this manuscript. (PDF)

S1 Fig *Ndr1* and *Ndr2* transcripts are broadly expressed in mouse embryos at E8.5. Whole mount *in situ* hybridization to *Ndr1* (top) and *Ndr2* (bottom) transcripts in wild-type embryos at E8.5. *Ndr1* and *Ndr2* expression was analyzed in three wild-type embryos each.
(TIF)

S2 Fig Wild-type and *Ndr1/2*-double null embryos show low and comparable levels of apoptosis at E8.5. Detection of apoptotic cells by the TUNEL method using FITC-labeled nucleotides in wild-type (left) and *Ndr1/2*-double null (right) embryo. Apoptotic cells are shown in green and DNA is stained in blue. Four wild-type and four *Ndr1/2*-double null embryos were analyzed.
(TIF)

S3 Fig YAP targets are expressed normally in *Ndr1/2*-double null embryos at E8.5. RNA was isolated from control and *Ndr1/2*-null embryos at E8.5 as described in Materials and Methods. Purified total RNA was used for gene expression analysis by qRT-PCR. Data shown represent the average transcript levels in three independent embryos per genotype. Each embryo was analyzed in triplicate. Statistical analysis was performed using a two-tailed T-test assuming unequal variance.
(TIF)

S4 Fig Mitotic index analysis of wild-type and *Ndr1/2*-double null embryos at E8.5. (A, B) At E8.5 mitotic cells in wild-type (A) and *Ndr1/2*-double null embryos (B) were visualized on paraffin sections with a specific anti-phospho Histone 3 antibody. Nuclei were counterstained by DAPI. (C) Quantification of the mitotic index in wild-type and *Ndr1/2*-double null embryos at E8.5. The mitotic index was defined as the percentage of phospho-H3 positive cells per embryo. Four embryos per genotype were analyzed with five independent sections per embryo being quantified. In total, more than 40,000 cells were analyzed per genotype. Error bars indicate the standard error of the mean (SEM).
(TIF)

S5 Fig Moderate increases of cell cycle duration over a short time period can potentially cause significant growth retardation of the mouse embryo at E8.5. Illustration of a mathematical model to approximate the effect of an increase in cell cycle duration on embryo growth at E8.5. The model is based on the simplifying assumption that all cells in the embryo divide at the same, constant rate t_wt_ from E7.5 to E8.5. This leads to an exponential equation where X_t_ = X_0_ * 2^t/t^. X_0_ is the total cell number of the embryo at t_0_, t is the cell cycle duration in hours and X_t_ the number of cells after t hours. We subsequently introduce the ratio “Q” of X_t, wild type_ over X_t, mutant_ to describe relative growth retardation as a function of the increase in t in the mutant. Q is plotted as a function of increasing cell cycle duration in the mutant after a period of 24 hours (t = 24). We observed earlier that wild-type embryos were approximately 1.5 fold bigger than *Ndr1/2*-double null littermates at E8.5 (Fig 3). According to our model, an increase of cell cycle duration from 6.7 hrs* to 8 hrs (intersection of dashed and solid red line) over 24 hours would suffice to generate a 1.5 fold difference at E8.5. *approximate cell cycle duration of 6.7 hrs at E7-7.5 was taken from *Snow*, *J*. *Embryol*. *Exp*. *Morphol* (1977) 42, 293–303.
(TIF)

S1 Table List of upregulated genes in *Ndr1/2*-double null embryos (at E8.5). Transcript levels were compared between control and *Ndr1/2*-double null embryos. Pooled transcripts obtained from three *Ndr1/2*-double null and three control littermate embryos were compared. Only genes whose expression is significantly altered are listed (p ≤ 0.05). A detailed description of the applied statistical analyses is provided in the Materials and Methods section.
(XLS)

S2 Table List of downregulated genes in *Ndr1/2*-double null embryos (at E8.5). Transcript levels were compared between control and *Ndr1/2*-double null embryos. Pooled transcripts obtained from three *Ndr1/2*-double null and three control littermate embryos were compared. Only genes whose expression is significantly altered are listed (p ≤ 0.05). A detailed description of the applied statistical analyses is provided in the Materials and Methods section.
(XLS)
