# Supplementary figures and images for: NDR Kinases Are Essential for Somitogenesis and Cardiac Looping during Mouse Embryonic Development
Source: PLoS One. 2015 Aug 25;10(8):e0136566. doi: 10.1371/journal.pone.0136566 (PMC4549247; doi:10.1371/journal.pone.0136566)

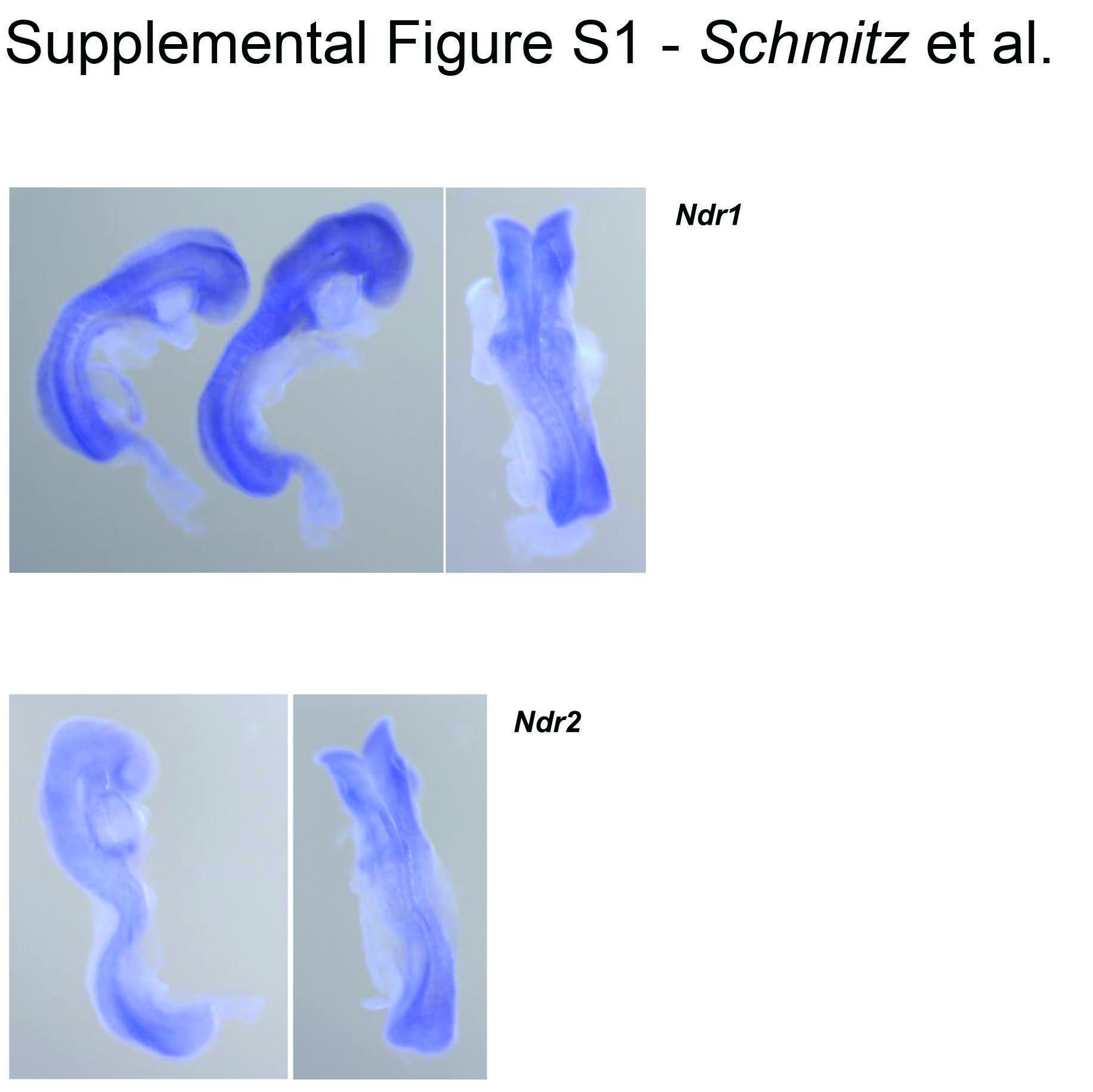

Supplement: S1 Fig — Whole mount in situ hybridization to Ndr1 (top) and Ndr2 (bottom) transcripts in wild-type embryos at E8.5. Ndr1 and Ndr2 expression was analyzed in three wild-type embryos each. (TIF) [file pone.0136566.s002.tif]

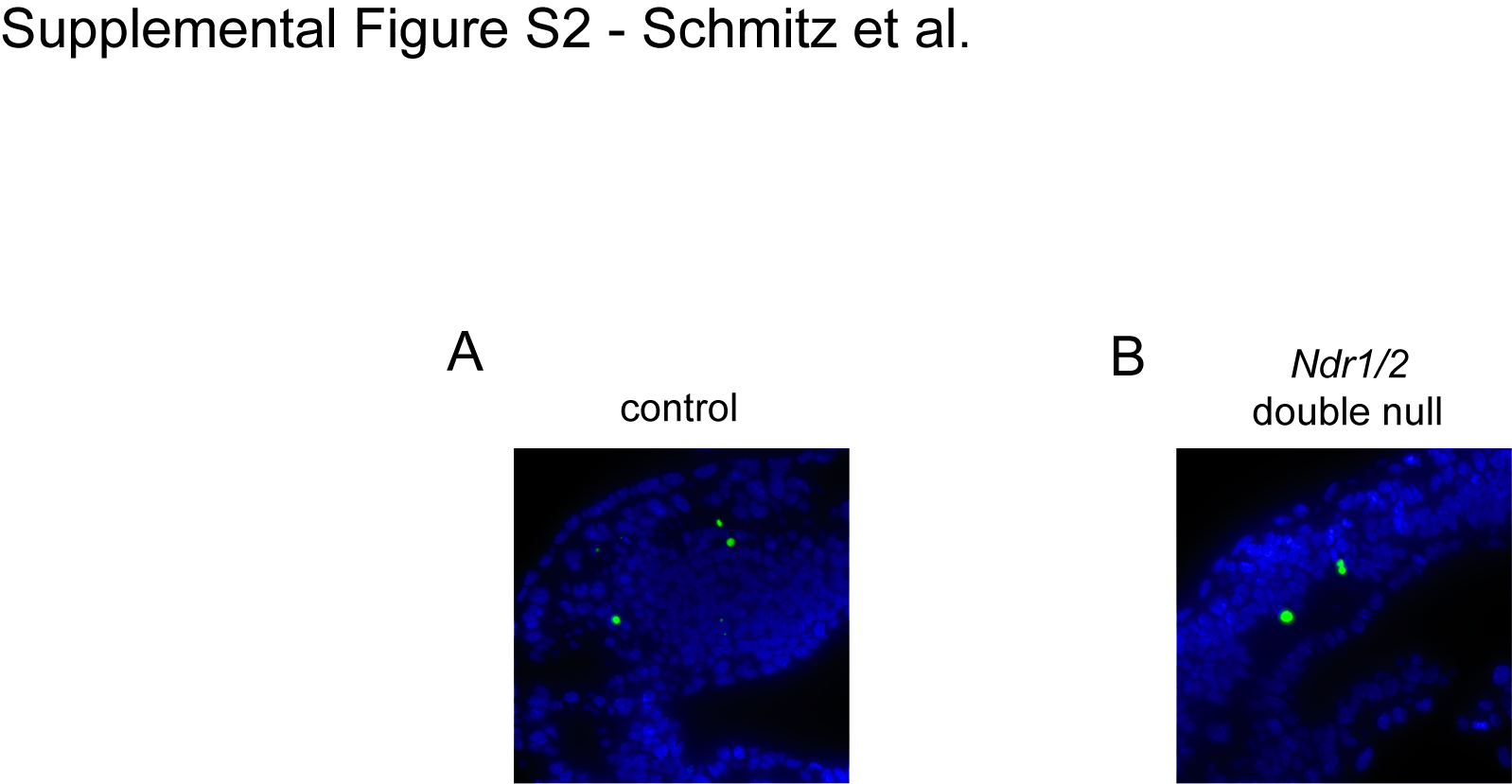

Supplement: S2 Fig — Detection of apoptotic cells by the TUNEL method using FITC-labeled nucleotides in wild-type (left) and Ndr1/2-double null (right) embryo. Apoptotic cells are shown in green and DNA is stained in blue. Four wild-type and four Ndr1/2-double null embryos were analyzed. (TIF) [file pone.0136566.s003.tif]

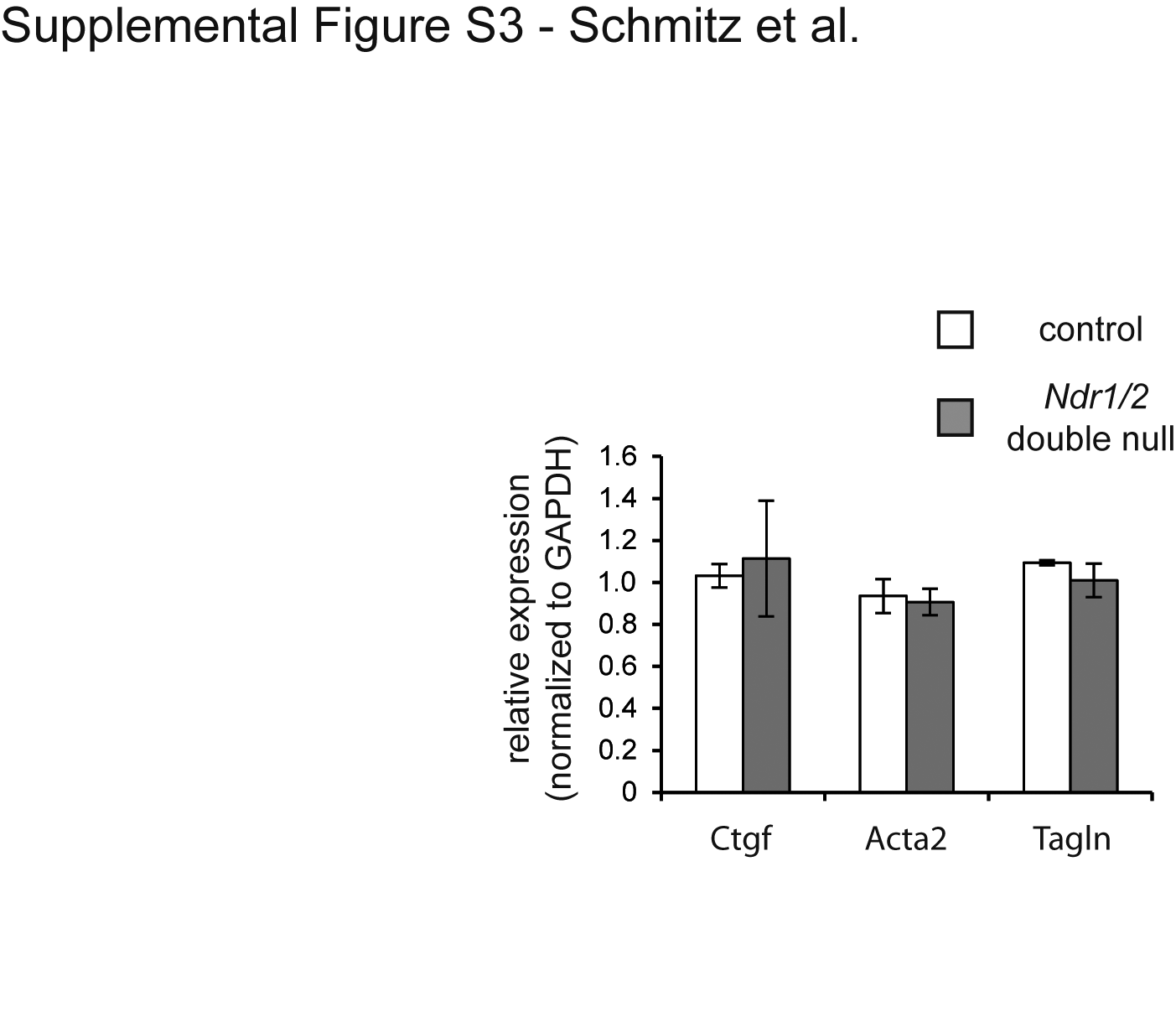

Supplement: S3 Fig — RNA was isolated from control and Ndr1/2-null embryos at E8.5 as described in Materials and Methods. Purified total RNA was used for gene expression analysis by qRT-PCR. Data shown represent the average transcript levels in three independent embryos per genotype. Each embryo was analyzed in triplicate. Statistical analysis was performed using a two-tailed T-test assuming unequal variance. (TIF) [file pone.0136566.s004.tif]

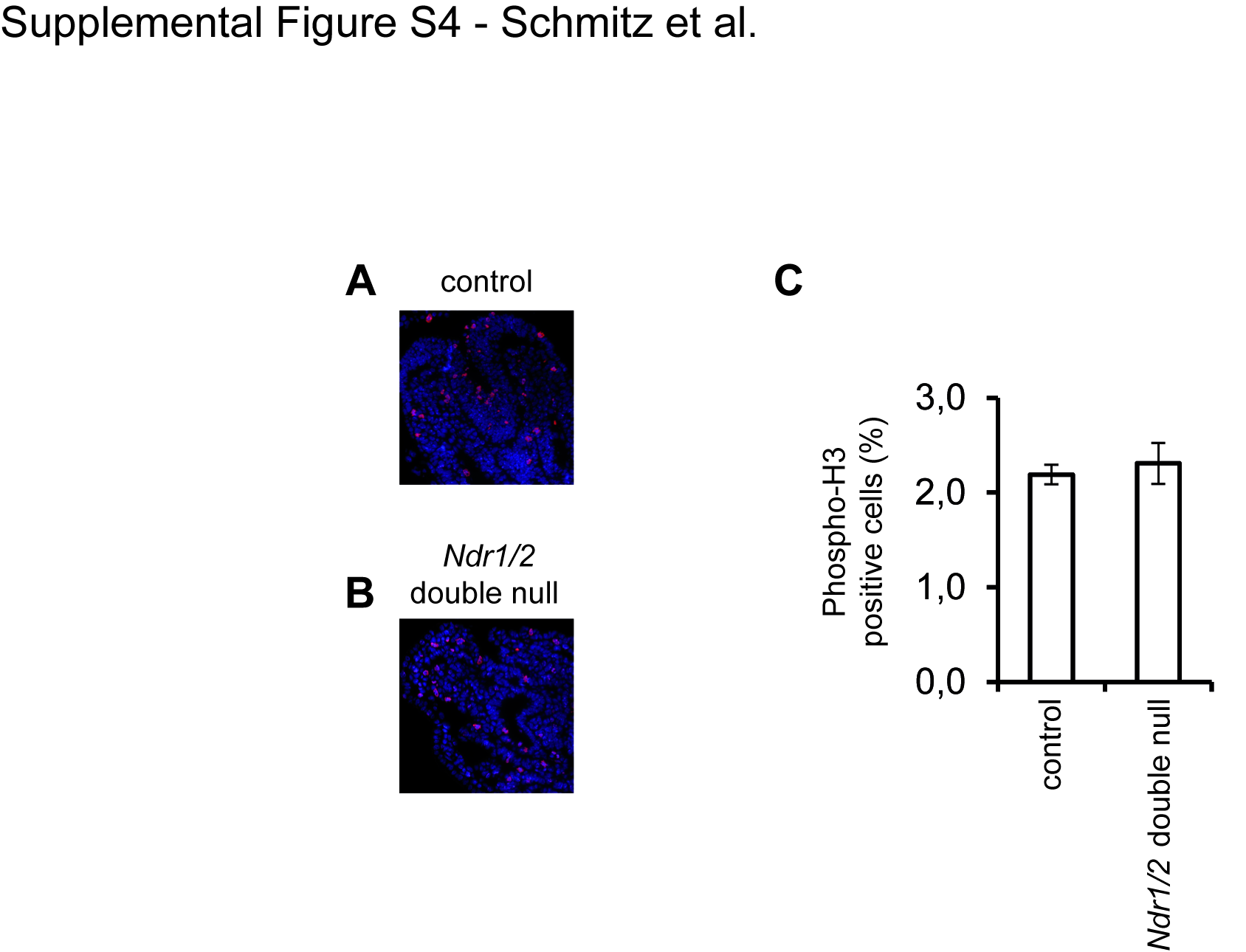

Supplement: S4 Fig — (A, B) At E8.5 mitotic cells in wild-type (A) and Ndr1/2-double null embryos (B) were visualized on paraffin sections with a specific anti-phospho Histone 3 antibody. Nuclei were counterstained by DAPI. (C) Quantification of the mitotic index in wild-type and Ndr1/2-double null embryos at E8.5. The mitotic index was defined as the percentage of phospho-H3 positive cells per embryo. Four embryos per genotype were analyzed with five independent sections per embryo being quantified. In total, more than 40,000 cells were analyzed per genotype. Error bars indicate the standard error of the mean (SEM). (TIF) [file pone.0136566.s005.tif]

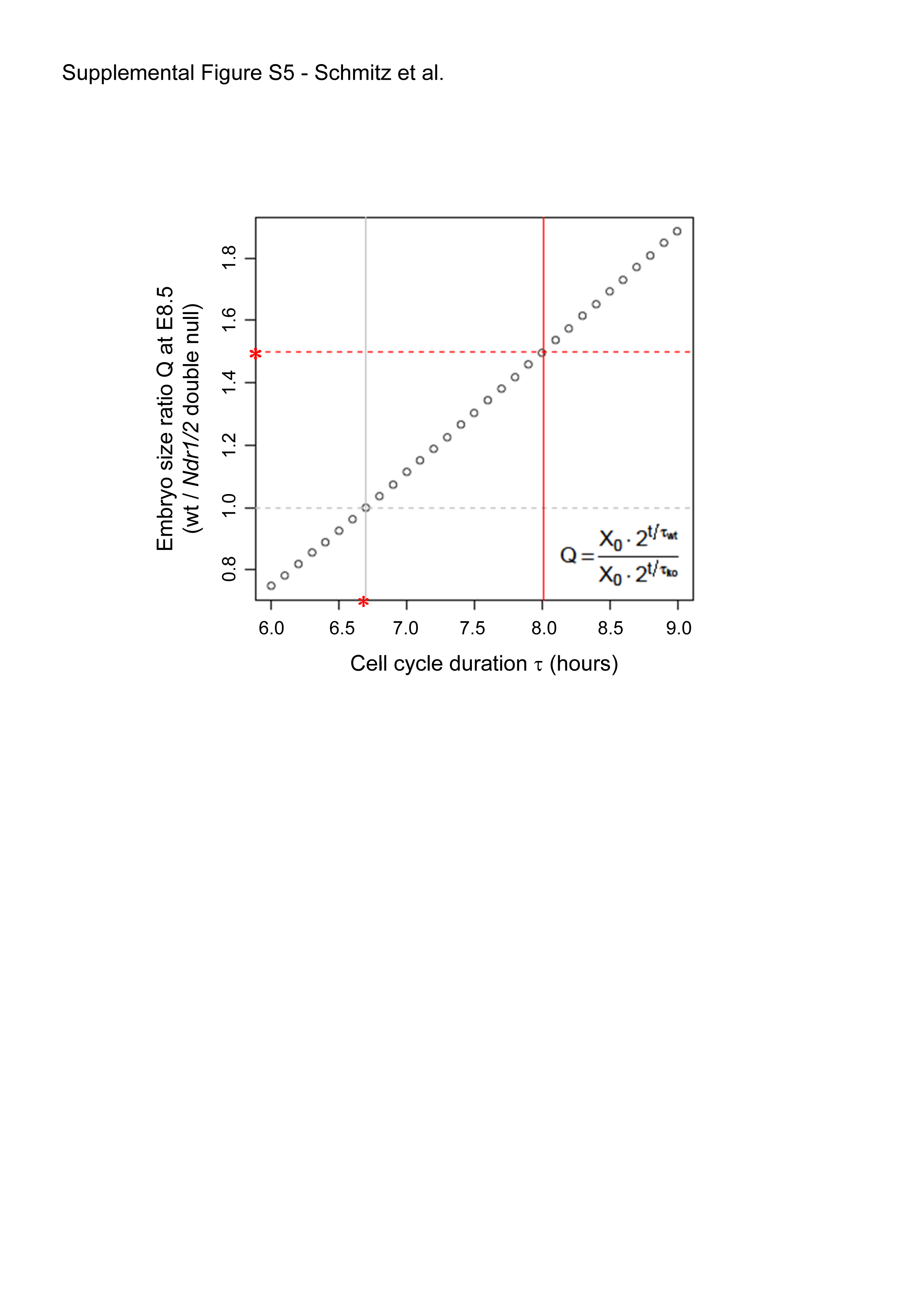

Supplement: S5 Fig — Illustration of a mathematical model to approximate the effect of an increase in cell cycle duration on embryo growth at E8.5. The model is based on the simplifying assumption that all cells in the embryo divide at the same, constant rate twt from E7.5 to E8.5. This leads to an exponential equation where Xt = X0 * 2t/t. X0 is the total cell number of the embryo at t0, t is the cell cycle duration in hours and Xt the number of cells after t hours. We subsequently introduce the ratio “Q” of Xt, wild type over Xt, mutant to describe relative growth retardation as a function of the increase in t in the mutant. Q is plotted as a function of increasing cell cycle duration in the mutant after a period of 24 hours (t = 24). We observed earlier that wild-type embryos were approximately 1.5 fold bigger than Ndr1/2-double null littermates at E8.5 (Fig 3). According to our model, an increase of cell cycle duration from 6.7 hrs* to 8 hrs (intersection of dashed and solid red line) over 24 hours would suffice to generate a 1.5 fold difference at E8.5. *approximate cell cycle duration of 6.7 hrs at E7-7.5 was taken from Snow, J. Embryol. Exp. Morphol (1977) 42, 293–303. (TIF) [file pone.0136566.s006.tif]
